# Revisiting of Cancer Immunotherapy: Insight from the Dialogue between Glycolysis and PD-1/PD-L1 Axis in the Tumor Microenvironment

**DOI:** 10.7150/ijbs.104079

**Published:** 2025-01-13

**Authors:** Qiong Liu, Zihan Liu, Xi Zhang, Anqi Zeng, Linjiang Song

**Affiliations:** 1 School of Medical and Life Sciences, Chengdu University of Traditional Chinese Medicine, Chengdu, China.; 2 Translational Chinese Medicine Key Laboratory of Sichuan Province, Sichuan Academy of Chinese Medicine Sciences, Sichuan Institute for Translational Chinese Medicine, Chengdu, Sichuan 610041, China.

**Keywords:** Glycolysis, PD-1/PD-L1, Lactate, Tumor microenvironment, immunotherapy

## Abstract

The interplay between metabolic pathways and immune escape has emerged as a captivating research area in oncobiology. Among these, the Warburg effect stands out as a hallmark metabolic reprogramming in cancer, characterized by elevated glucose utilization and excessive lactic acid production through anaerobic glycolysis. Key glycolytic enzymes not only fulfill the bioenergetic demands of cancer cells but also exhibit moonlighting roles, including regulation of epigenetic modifications, protein kinase activity, and immune escape mechanisms, thereby reshaping the tumor microenvironment. Tumor-specific vascular architecture facilitates lactate accumulation, which drives tumor progression by impairing immune cell function and acting as a signaling molecule to recruit immunosuppressive cells and modulate immune checkpoint pathways. The PD-1/PD-L1 co-stimulatory pathway plays a crucial role in negatively modulating the activation, proliferation, and cytokine secretion by T-lymphocytes. This review primarily focuses on elucidating the regulation and mechanisms underlying PD-1/PD-L1 signaling axis during glycolysis in tumor cells as well as surrounding cells. In the era of precision medicine, there is a particular interest in leveraging ^18^F-FDG PET/CT imaging as a valuable tool to assess PD-L1 expression status for more targeted therapeutic interventions. Additionally, the development of natural compounds capable of modulating metabolism opens new avenues for metabolism-based immunotherapy, though further studies are required to validate their *in vivo* efficacy.

## Introduction

Cancer immunotherapy harnesses the immune defenses of host to reinvigorate and sustain the cancer-immunity cycle, thereby combating the progression of various malignancies. Over the past decades, groundbreaking advances have been made through immunotherapy modalities, including immune checkpoint inhibitors (ICIs), adoptive cell therapies, and tumor vaccines^1^. Among these, immune checkpoint blockade (ICB) therapy, particularly anti-PD-1/PD-L1 treatments, has marked a milestone in anticancer strategies^2^. These therapies have substantially improved the prognosis of patients with advanced and refractory cancers^3^. A realistic benefit of ICB is their ability to elicit prolonged remissions in certain cancers, with patients responding to PD-1/PD-L1 inhibitors often experiencing stable disease for extended periods, in stark contrast to the typically transient and recurrent effects of chemotherapy. Despite these breakthroughs have been transformative, the clinical application of PD-1/PD-L1 inhibitors is far from straightforward. Several limitations, including resistance development, limited efficacy in "cold" tumors, immune-related adverse events, and the absence of reliable biomarkers for patient selection, continue to impede the full realization of the immunotherapeutic potential of ICB^4^. Nonetheless, ongoing research into combination therapies, novel biomarkers, and ways to modulate the TME is poised to refine and expand the use of these ICIs, potentially accrue better outcomes for a broader patient population.

The dual metabolic reprogramming occurring in tumor and immunological cells constitutes a pivotal aspect influencing the anti-tumor immune response in TME^5^,^6^. Tumor cells exhibit a preference for rapid energy acquisition through the Warburg effect to support their high proliferative rate while reducing oxidative stress. Additionally, they generate excessive lactate, which further affects phenotypic and functional characteristics of immune cells^7^. Similarly, immune cells undergo distinct metabolic patterns during their transition from homeostasis to activation, which significantly impacts their proliferation, differentiation, and effector function^8^. Due to the mismatch between energy demand and availability of nutrients, cancerous cells and immune cells engage in metabolic competition, which hinders the full potential of immune responses and accelerates tumor growth. Key enzymes involved in glycolysis have a dual role, supporting efficient metabolism in tumor cells while also regulating tumor occurrence and development through non-metabolic mechanisms like anti-apoptosis, immune escape mediation, and participation in signaling pathways^9^. In addition to causing cellular acidosis, lactate in the TME serves as an energy substrate that shuttles between various cell populations comprising neoplastic cells, tumor-infiltrating lymphocytes, tumor-associated macrophages(TAM) and tumor-associated stromal cells^10^.Researchers have also gradually recognized that the metabolite lactate, as a signaling molecule, mediates cross-talk between metabolic pathways, immune responses, and intercellular communication within the TME^7^,^11^. The metabolic barriers to effective antitumor immunity create a vicious cycle where immune suppression fuels further tumor growth, and tumor progression, in turn, exacerbates the metabolic constraints. As such, targeting the metabolic pathways involved in glycolysis represents a promising therapeutic strategy to reprogram the TME and restore immune function.

In this context, recognizing the interdependent relationship between hyper-glycolysis and tumor immune escape is critical^12^,^13^. This review seeks to elucidate the intricate connections between the PD-1/PD-L1 axis and glycolytic metabolism within the TME, homing in on how glycolytic enzymes and lactate regulate this axis and its implications for immunotherapy. By elucidating the nexus between energy metabolism and immune function, we propose that targeted manipulation of both glycolytic pathways and the PD-1/PD-L1 axis could enhance anti-tumor immune responses, offering new therapeutic strategies for cancer treatment.

## Influence of PD-1/PD-L1 axis and glycolysis on tumor immunity within the TME

The PD-1 and its ligand(PD-L1) belong to the B7 family of co-stimulatory molecules, also known as the “molecular brake” *in vivo*. PD1(CD80) is a type I transmembrane glycoprotein encoded by the PDCDI gene, consisting of 288 amino acids^14^. The cytoplasmic tail harbors an immune-receptor tyrosine-based inhibitory motif(ITIM) as well as an immune-receptor tyrosine-based switch motif(ITSM). Activated monocytes and dendritic cells(DC), T cells, B cells, and natural killer(NK) cells all express PD-1, while resting T cells lack PD-1 expression but can upregulate it upon activation. PD-1 recognizes two ligands, namely PD-L2(CD273 or B7-DC) and PD-L1(CD274 or B7-H1), which are also type I transmembrane proteins comprising of 270 and 290 amino acids, respectively. PD-L1 is extensively present in several cell types, such as T cells, B cells, macrophages, dendritic cells and tumor cells^3^. Unlike PD-L1, the expression profile of PD-L2 is narrow and limited to antigen presenting cells. Notably, up-regulation of PD-L1 significantly contributes to the promotion of immune evasion by malignancies. It has been noted in clinical practice that patients with elevated PD-L1 expression on tumor cells or a substantial infiltration of lymphocytes in the tumor are more likely to benefit from it^2^. As a result, we ponder upon the characteristics of patients exhibiting highly expressed PD-L1 molecules and the molecular mechanisms within tumor cells that induce such high expression. The TME impacts the success or failure of immunotherapy. In addition to immune suppression, metabolic factors within the TME can limit the effectiveness of PD-1/PD-L1 blockade. Therefore, it becomes imperative to revisit the fundamental attributes of malignancy.

**Figure 2:**
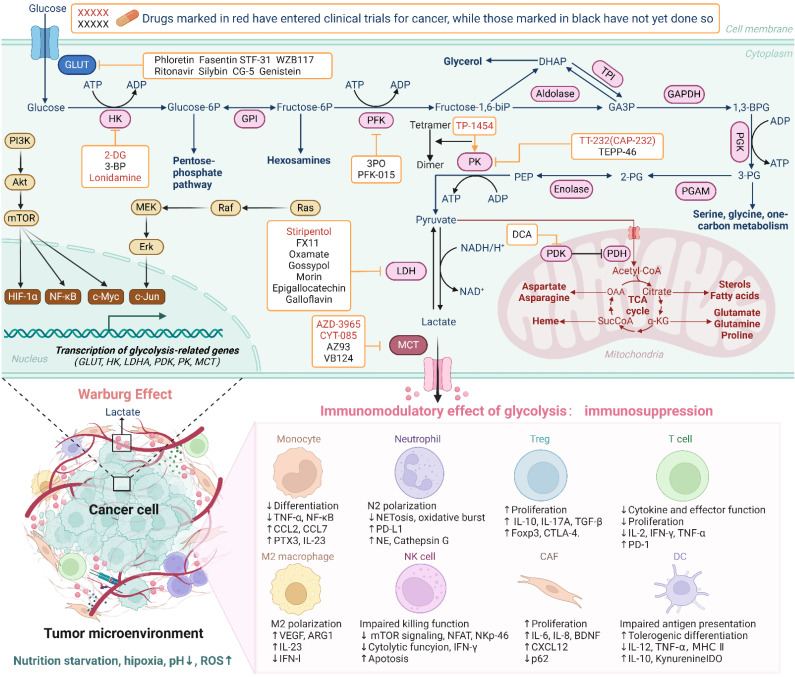
Overview of glycolysis in cancer cells and its immunomodulatory effects on immune cells within the TME. In cancer cells, glucose is rapidly transported by GLUT, converted into pyruvate through key glycolytic enzymes, then into lactate by LDH, and transported to the TME by MCT, bypassing oxidative phosphorylation. This metabolic shift has significant immunomodulatory effects. Elevated lactate levels and other metabolic byproducts can create an acidic TME, which impairs the function of immune cells. High lactate concentrations inhibit the activation and proliferation of cytotoxic T cells, promote the differentiation of immunosuppressive Tregs, and affect macrophage polarization, dendritic cells, neutrophil and other immune cell subsets, thereby fostering an immunosuppressive environment that supports tumor progression. (Created with Biorender.com).

Altered metabolism is a crucial hallmark of tumor cells, with the "Warburg effect" being the most renowned theory in tumor metabolism^15^. Hyper-glycolytic traits in tumor cells are exactly co-driven by oncogene activation, convoluted signaling pathways, high metabolic demands and the anoxic TME^16^. These factors contribute to increased glucose uptake, enhanced glycolytic enzyme activity, and elevated lactate production (Figure 2). The TME, comprising tumor cells, tumor-infiltrating monocytes/macrophages, immunosuppressive cells, fibroblasts, vasculature, and the inflammatory mediators they secrete, engages in a metabolic "dialogue." This interaction influences the metabolic activities of the cells themselves, thereby defining specific roles within the TME. A salient feature of the TME is its dynamic evolution, reflecting the changing metabolic states of its constituent cells. During the initial stages of tumorigenesis, the TME manifests as an immune-promoting milieu with heightened pro-inflammatory signals, promoting pro-inflammatory phenotypes in immune cells. As tumors proliferate and metastasize, the TME gradually shift towards an immunosuppressive milieu typified by low oxygen, acidic pH, diminished glucose levels, elevated fatty acids, reduced amino acids, heightened adenosine, and increased lactate. Lactate accumulation directly affect immune cells: suppresses cytotoxic T lymphocyte and natural killer (NK) cell activity, inhibits pro-inflammatory cytokine production (e.g., IFN-γ, TNF-α), and promotes regulatory T cell (Treg) expansion. Suppression of DC function, infiltration and polarization of macrophages toward an M2-like phenotype, and tumor-associated neutrophils(TANs) toward an N2-like phenotype^5^,^17^. The metabolic shift towards glycolysis also impacts the vascularization and nutrient availability within TME. Undue glucose consumption by cancer cells results in local hypoxia, which further promotes the glycolytic switch and the production of additional immunosuppressive factors like hypoxia-inducible factor 1-alpha (HIF-1α)^7^. HIF-1α, a key regulator of the hypoxic response, not only boosts glycolytic activity but also stimulates the expression of PD-L1 on both tumor and stromal cells^18^. In this complex and dynamic TME, various non-tumor cells exhibit intricate and shifty functions^19^. Immune cells, for example, differentiate into distinct phenotypes, metabolic profiles, and functional subgroups, which either oppose or support tumor growth, depending on the local milieu. These findings imply that glycolytic reprogramming confers both an endogenous growth advantage to tumor cells and an extrinsic effect of immune dysfunction. As a result, key enzymes and transporters of the tumor glycolysis pathway serve as important targets for the development of tumor immunotherapy drugs(Figure 2).

## Whispers Between the glycolysis and the PD-1/PD-L1 axis in TME: Unraveling the Secrets of Tumor Immunity Escape

### GLUT1 and PD-1/PD-L1 axis

The primary energy source for cancerous cells is glucose, and the first rate-determining step in glycolysis is the transfer of glucose across the plasma membrane^20^. In addition to the high proliferation index observed in tumorigenic cells, research indicates that elevated GLUT1 expression is associated with poorer survival and a weak response to adjuvant immunochemotherapy. A retrospective research has showcased that radiotherapy possesses the capacity to augment the manifestation of PD-L1 within hypopharyngeal cancer cells, plausibly contingent upon GLUT1 expression^21^. Meanwhile, radiotherapy also makes it easier for CD4+ and CD8+ T cells to infiltrate cancerous tissues, creating a favorable environment for PD-1/PD-L1 blocking. Currently, research on metabolic reprogramming and its interplay with tumor immunity following radiotherapy remains scarce. Inactivation of GLUT1 enhances the sensitivity of tumor cells to TNF-α-induced anti-tumor immunity by elevating ROS levels^22^. The function of cytotoxic T cells, which expresses GLUT3 at high levels, is not significantly affected by inhibiting GLUT1^23^. Combining GLUT1 inhibitor BAY-876 with anti-PD-1 treatment leads to a significant increase in the number of infiltrated CD45+ immune cells^22^. Li *et al.* develop a strategy that combines the GLUT1 inhibitor BAY-876 with the PD-L1 inhibitor BMS-1, delivered via an injectable thermogel formulation, fostering sustained antitumor immunity and improved survival outcomes in glioblastoma models^24^. Based on scRNA-seq data and immunofluorescence, Liu *et al.* identified a specific enrichment of gap junction protein beta-2 (GJB2) in hepatocellular carcinoma(HCC) cells, with a tendency for it to localize primarily in the cytoplasm^25^. Through facilitating the ubiquitination and degradation of IκBα, GJB2 activates the NF-κB pathway, which subsequently initiates the HIF-1α/GLUT-1 axis tied to glycolytic processes, culminating in increased PD-L1 expression. The NAT10-catalyzed N4-acetylcytidine modification in cervical cancer cells upregulates FOXP1, which promotes GLUT4 and KHK expression, supporting a glycolysis-shaped immunosuppressive TME^26^. Tellingly, NAT10 loss improved the efficacy of PD-L1 blockade therapy.

Nerve growth factor (NGF) secretion from non-small cell lung cancer(NSCLC) cells promotes neuronal differentiation and axon formation within the TME. Nerve fibers in the TME release 5-hydroxytryptamine (5-HT), which binds to NSCLC cells, activating the HTR1D receptor. This activation enhances glycolytic activity through PI3K/Akt/mTOR pathway, which promotes immune evasion by increasing PD-L1 expression and decreasing major histocompatibility complex (MHC) levels^27^. Importantly, inhibiting the tumor immunometabolic reprogramming driven by 5-HT with 2-DG has been shown to improve the effectiveness of anti-PD-1 therapy in mouse models. Citalopram, selective serotonin reuptake inhibitors, recently was found combat HCC by inhibits GLUT1^28^. This inhibition leads to a reduction in glycolytic flux and ATP production necessary for tumor growth. Additionally, mutations at the citalopram binding site on GLUT1 compromises its efficacy. Preclinical models of HCC reveal that citalopram not only slows tumor growth but also enhances the efficacy of anti-PD-1 therapy, suggesting a synergistic effect.

### HK and PD-1/PD-L1 axis

**Figure 3:**
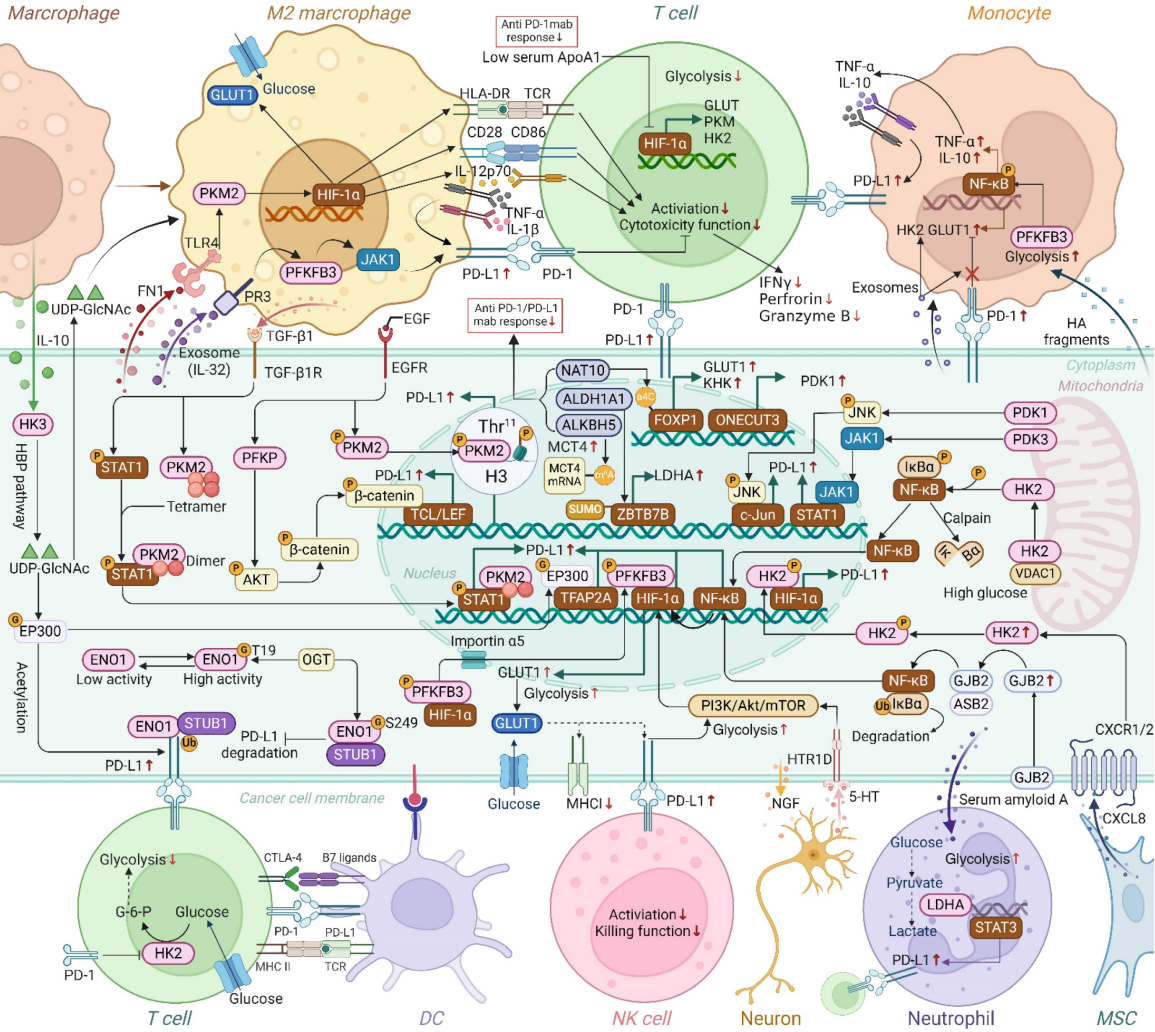
Crosstalk of glycolytic genes and PD-1/PD-L1 in TME. Glycolytic enzymes and transporters have been shown to participate in the upregulation of PD-L1 in cancer cells through mechanisms such as enhanced transcription, ubiquitination, histone phosphorylation and glycosylation, a process that involves crosstalk between different cell types. Additionally, glycolytic enzymes in macrophages, monocytes, T cells, and neutrophils have been found to regulate the expression of PD-L1 or PD-1. Thus, beyond their classical metabolic roles, glycolytic enzymes are increasingly recognized for their involvement in promoting immune evasion. Moreover, the regulation of glycolysis-related genes(NAT10, ALDH1A1, ALKBH5, ApoA1, ONECUT3, etc.) is closely associated with diminished responses to anti-PD-1/PD-L1 therapies. (G, O-GlcNAcylation; P, phosphorylation, Ub, ubiquitination; M^6^A, N6-methyladenosine; SUMO, SUMOylation; HBP, hexosamine biosynthesis). (Created with Biorender.com).

Human tissues express four hexokinase isoforms (HK1-4) that catalyze the conversion of glucose to glucose-6-phosphate (G-6-P), initiating glycolysis. The N-terminal hydrophobic domain of HK1 and HK2 binds to the voltage-dependent anion channel (VDAC) on the mitochondrial outer membrane, providing a "kinetic advantage" for the hexokinase reaction^29^,^30^. Under high glycolytic conditions, the excess G-6-P produced by HK2 can cause conformational changes, resulting in the dissociation of HK2 from the mitochondrial outer membrane. HK1 binds strongly to mitochondria to support glucose metabolism, whereas HK2, with its fluctuating affinity for VDAC, moves between the cytoplasm and mitochondria, adeptly adjusting to environmental and metabolic fluctuations^31^. According to Guo *et al.*, HK2 can also function as a protein kinase, activating IκBα, leading to its degradation and an increase in PD-L1 expression through NF-κB activation, aiding tumor immune evasion(Figure 3)^32^. Additionally, concomitant use of HK inhibitors with PD-1 antibodies significantly improves their therapeutic efficacy in treating mouse glioma. This study clarified that HK2, as a glucose receptor, can sense intracellular glucose level and link energy metabolism with tumor immunity through the conversion of classical and non-classical functions, revealing the mechanism of Warburg effect promoting tumor immune escape. Similarly, Lin *et al.* found that high glucose levels increase PD-L1 expression in breast cancer cells via HK2, contributing to immune-privileged status^33^. In turn, previous study indicate that PD-L1^high^ tumors outcompete neighboring T cells by creating a metabolically harmful environment through HK2-enhanced glycolysis^34^. HK3 enhances the stability and activity of EP300 through O-GlcNAcylation at Ser900, preventing its degradation and promoting its function in PD-L1 transcription alongside TFAP2A^35^. Additionally, the interaction between HK3 and TAMs is highlighted by the influence of IL-10 from M2 TAMs on HK3 expression. This interaction further facilitates the polarization of TAMs through the metabolic substrate uridine 5′-diphospho-N-acetylglucosamine (UDP-GlcNAc). Understanding HK3's role in modifying EP300 and influencing immune responses provides a promising target for improving the effectiveness of ICB therapies.

IL-8 from gastric cancer mesenchymal stem cells (GCMSCs) facilitates HK2 translocation to the nucleus, where phosphorylated HK2 interacts with HIF-1α to promote PD-L1 transcription^36^. GCMSCs also produce excess lactate, impairing CD8+ T cell function. Blocking the IL-8/CXCR2/HK2 axis effectively reduces PD-L1 expression and lactate production, enhancing the impact of anti-PD-1 therapy for advanced gastric cancer, with potential clinical benefits. The VEGFC-VEGFR3 and FGF2-FGFR1 signaling pathways orchestrate lymphangiogenesis and immune evasion in cholangiocarcinoma by enhancing lactic acid production via HIF-1α and c-MYC-mediated HK2 expression, and regulating PD-L1 expression via the NF-κB/Cox-2 pathway^37^. The simultaneous blockade of FGFR and VEGFR attains a notable and comprehensive suppression of lymphangiogenesis and PD-L1 expression.

### PFK and PD-1/PD-L1 axis

Phosphofructokinase 1(PFK-1), as one of the important rate-limiting enzymes in glycolysis, is responsible for catalyzing the conversion of fructose-6-phosphate(F-6-P) to fructose-1, 6-diphosphate(F-1,6-P). Phosphofructokinase-2(PFKFB) can catalyze F-6-P to fructose-2, 6-diphosphate, which is the strongest allosteric activator of PFK-1 and an effective stimulator of glycolysis. According to different configurations, PFK-1 is divided into three subtypes, namely liver PFK, muscle PFK, and platelet PFK(PFKP). Among the four subtypes of PFKFB, PFKFB3 possess a distinctly higher kinase/phosphatase activity ratio, rendering it a crucial enzyme in regulating the glycolytic flux of tumor cells^38^. Based on the experimental results, PFKFB3 inhibitors showed significant inhibitory effect in tumors. However, surprisingly, satisfactory tumor suppression was not observed in clinical trials^39^. Zheng *et al.* discovered that the application of the inhibitor PFK-015 can stimulate phosphorylation of PFKFB3 at the Ser461 residue, thereby augmenting its interaction with HIF-1α, and that they co-localize in the nucleus^40^. This process results in HIF-1α transcriptional upregulation of PD-L1 expression, further promoting tumor immune evasion, which mitigates the cytotoxic effect of selective PFKFB3 inhibitors. Administration of combined ICIs, such as anti-PD-1 mAb, might be considered to compensate for this effect. IL-32γ, generated by myeloma cells, potentiates PFKFB3 reliant glycolysis via protease 3, facilitating the activation of the PFKFB3-JAK1 signaling axis, which promotes macrophage expression of PD-L1 and impedes CD8+T cell function to foster the progression of multiple myeloma^41^. Monocytes that have been stimulated emit autocrine TNF-α and IL-10, which stimulate the PD-L1 level on monocytes themselves. PD-L1+monocytes efficiently facilitate human tumor growth and quell tumor specific T cell immunity. Soluble factor hyaluronic acid, derived from tumors, induce upregulation of PFKFB3 in tumor-associated monocytes, which promotes PD-L1 expression by activating NF-κB signaling^42^. The findings underscore the regulatory role of PFKFB3 in the oncogenic properties of monocytes within the TME. The adaptive changes of low glucose in tumor microenvironment of renal cancer cells regulate associated life activities and provoke the up-regulation of key enzymes in glycolysis. The glycolysis level is still low due to the lack of glucose. On the other hand, low glucose activates EGFR to promote cell proliferation and mediates immune escape by up-regulating the PD-L1 level via the EGFR/ERK/c-Jun pathway^43^. In turn, the increased PD-L1 regulates glycolysis through the feedback of PFKFB3 to avoid glycolytic overcapacity. PFKP has been discovered to have evident immunomodulatory effects in many cancers, and its diagnostic value is founded upon its positive association with PD-L1^44^. PFKP amplify the Warburg effect and elevate the PD-L1 expression through AKT activation, promoting the progression of glioblastoma^45^,^46^. EGFR activation triggers PFKP to bind to EGFR, leading to phosphorylation of PFKP Y64, which up-regulates PD-L1 expression through AKT-mediated phosphorylation of β-catenin S552^45^.

### PK and PD-1/PD-L1 axis

Pyruvate kinase (PK) transfers a phosphate group from phosphoenol pyruvate to ADP to generate pyruvate and ATP, culminating the ultimate rate-determining stride of glycolysis^47^. Amongst the quartet of PK subtypes, it is PKM2 that assumes a significant function in preserving tumor cell proliferation, metastasis, and anabolic processes. Recent investigations have highlighted how PKM2 impact immune function beyond its promotion of oxidative phosphorylation and Warburg effect, establishing a crucial link between energy metabolism and tumor immunity^48^. Guo *et al.* discovered through cellular experiments that PKM2 potentiates the augment of PD-L1 expression in lung adenocarcinoma cells, portending an unfavorable prognosis for patients^49^. Similarly, another study also revealed the synergistic effect of PKM2 and PD-L1 on tumor cells and immune cells in lung adenocarcinoma tissue as a powerful prognostic factor for lung adenocarcinoma patients^50^. PKM2 exists in two configurations: an active tetrameric form and a less active dimeric form. In cancerous cells, the dimeric variant is more prevalent, leading to an accumulation of glycolytic intermediates. This accumulation not only satisfies the energy requirements of the tumor but also provides precursor molecules for biosynthetic pathways that support rapid cellular proliferation. When existing as a dimeric entity, PKM2 can also penetrate the nucleus as a co-transcriptional factor and protein kinase, coordinating the modulation of gene transcription and contributing to the progression of tumorigenesis^51^. The nuclear PKM2 stimulates the function of the reverse transcriptional activation domain of HIF-1α, and binds the hypoxia response element of the HIF-1α target gene recruited by P300 to the HRE site on the PD-L1 promoter, thereby upregulating the expression of PD-L1 on TAM, DC, T cells, and CT26 colon cancer cells. The allosteric activator of PKM2 (TEPP-46) can impede the PD-L1 expression on LPS-induced immune cells and cancerous cells, effectively thwarting tumor immune evasion^52^. Li et.al further discovered that high PKM2 expression not only fosters an inflammatory and immunosuppressive TME but also correlates with increased efficacy of PD-L1 blockade therapy in HCC patients^53^. Wang *et al.* demonstrated that epidermal growth factor (EGF) stimulates the phosphorylation of PKM2 Ser37, subsequently inducing histone H3 phosphorylation at T11 site, leading to chromatin relaxation and PD-L1 transcription upregulation in HCC cells^54^. Activation of PKM2, in its tetrameric form, results in a decreased release of lactate. The initial dosing of TP-1454, a small-molecule PKM2 activator, has occurred in a Phase 1/1b trial. The main aims of this first-in-human, open-label study are to evaluate the safety of once-daily oral TP-1454 as a monotherapy in patients with advanced metastatic or progressive solid tumors, as well as in combination with ipilimumab and nivolumab^55^.

The fibronectin 1(FN1) derived from HCC cells regulates the glycolytic metabolism of macrophages, which in turn affects their anti-tumor properties and the expression of PD-L1 mediated by inflammation^56^. Specifically, the binding between FN1 and TLR4 activates the PKM2/HIF-α axis, promoting the glycolysis of macrophages. On one hand, this glycolytic metabolism stimulates the high expression of CD86 and HLA-DR and the secretion of the anti-tumor cytokine IL-12P70, thereby enhancing the macrophages' anti-tumor properties. On the other hand, glycolytic metabolism fosters the PD-L1 expression on macrophages by causing the secretion of pro-inflammatory cytokines TNF-α and IL-1β, leading to tumor immune escape. However, the application of PD-L1 inhibitors can counteract the immune escape facilitated by PD-L1+ macrophages and unleash their intrinsic anti-tumor ability^56^. A study has shown that PKM2 nuclear translocation in sepsis further controls neutrophil PD-L1 in a STAT1-dependent manner^57^. TAM-derived TGF-β1 induces PKM2 nuclear translocation, which further motivates the deactivation of PD-L1 in pancreatic ductal adenocarcinoma(PDAC) cells through the JAK-STAT1 pathway, ultimately leading to inactivation and dysfunction of NK cells(Figure 3). Be consistent with, in vivo experiments have indicated that PKM2 knockdown and PD-1/PD-L1 blocking can jointly reverse the immune escape mechanism of PDAC, providing a novel target for the immunotherapy of PDAC^58^. TGF-β secreted by M2 TAM enhances glycolysis and facilitates immune evasion in bladder cancer by promoting the nuclear translocation of PKM2, which interacts with p-STAT3 to elevate PD-L1 levels, while dual targeting of TGF-β receptors and PKM2 shows significant potential in inhibiting tumor progression and enhancing immune responses^59^. In esophageal squamous cell carcinoma, the administration of the estrogen-related receptor gamma(ESRRG) specific agonist DY131 downregulate PKM2 expression with a marked suppression of glycolytic activity and lactate production, synergistically augmenting the efficacy of anti-PD-1 treatment^60^. HNRNPL enhances the stability of circFAM13B, which subsequently binds to IGF2BP1, leading to the destabilization of PKM2 mRNA. This interaction results in decreased PKM2 protein levels, thereby impairing glycolytic activity and reducing lactic acid in the TME^61^. Overexpressing CircFAM13B resulted in a prominent enhancement of CD8+ T cell activity and improved outcomes for anti-PD-1 therapy. Similarly, CircRHBDD1 recruites YTHDF1 to m6A-modified PIK3R1 mRNA, activating the glycolysis through PI3K/AKT pathway, and promoting resistance to anti-PD-1 therapy^62^.

Recent findings indicate that deletion of PKM2 in CD8+ T cells augments the pentose phosphate pathway, consequently increasing TCF1+ progenitor-like properties and reducing glycolytic dependence. This shift supports sustained functional longevity and bolsters T cell responsiveness to PD-1 blockade^63^. Low apolipoprotein A1 (ApoA1) serum levels impair CD8+ T cell cytotoxicity by diminishing HIF-1α-mediated glycolysis. Conversely, higher ApoA1 levels may enhance anti-PD-1 treatment efficacy and inhibit tumor progression by fostering a favorable immune environment for CD8+ T cells^64^.

### PDK and PD-1/PD-L1 axis

Pyruvate dehydrogenase kinase(PDK) shifts pyruvate metabolism from mitochondrial oxidative phosphorylation to the cytoplasm for glycolysis by suppressing the activity of the mitochondrial pyruvate dehydrogenase complex^65^. The control of PDK directly influences the efficiency of glycolysis, and its expression is often up-regulated in various cancers, correlating with tumor cell proliferation, metastasis, and poor prognosis^66^. In ovarian cancer, CD8+ T cell infiltration is negatively correlated with PDK1 expression, whereas PDK1 expression shows a positive correlation with PD-L1 levels. Elevated PDK1 levels up-regulate PD-L1 via the JNK-c-Jun pathway, thereby impairing CD8+T cell functionality, reducing IFN-γ secretion, and fostering apoptosis^67^. Liu *et al.* uncover that PDK3 increases PD-L1 expression in colorectal cancer(CRC) by activating the JAK1/STAT1 signaling pathway, while also promoting tumor proliferation through the PI3K/AKT signals^68^. On the contrary, in a study of 400 gastric cancer patients, Zhang et.al found that that low levels of PDK2 and PDK4, key glycolysis-related genes, correlated with lower PD-L1 expression and enhanced response to immune checkpoint blockade therapy, suggesting an inhibitory role on PD-L1 by the PDK family^69^. This new research also demonstrates that inhibiting PDK leads to an increase in PD-L1 expression on gastric cancer cells. And this upregulation is associated with enhanced histone acetylation, which is mediated by elevated levels of acetyl-CoA resulting from the inhibition of glycolysis. Despite the fact that the uncovered mechanisms of these experiments are different and the relationship between PDK and PD-L1 is inconsistent, intriguingly, they both demonstrate co-treatment with the PDK inhibitor and anti-PD-L1 antibody in vivo show better anti-cancer effect^67^-^69^. As a link between metabolism and immunity, the transcription factor ONECUT3 not only enhances aerobic glycolysis by transcriptionally regulating PDK1 but also controls CD8+T cell infiltration in pancreatic cancer. ONECUT3 knockdown or PDK1 inhibition can raise the effectiveness of anti-PD-1 treatment in PDAC^70^.

### LDH and PD-1/PD-L1 axis

Within the lactate dehydrogenase(LDH) family, LDHA subunit primarily responsible for the conversion of pyruvate into lactic acid, while its counterpart, LDHB, exerts an inverse effect. Lactate dehydrogenase assumes paramount importance in the preservation of tumor cell metabolism, enabling adaptability to adverse environmental conditions and cellular stress, as well as governing the intricate processes of tumor cell apoptosis and autophagy^71^,^72^. Moreover, recent investigations have accentuated the regulatory function of LDHA in tumor immune response^73^,^74^. K. Urata *et al.* have identified a significant relationship between PD-1/PD-L1 expression and LDHA in extramammary Paget's disease^75^. The inactivation of LDHA has been discovered to impede tumorigenesis and progression within tumor mouse models, underscoring the crucial role played by LDHA in promoting K-RAS induced primary breast cancer in mice^76^. Intriguingly, following the knockout of LDHA in macrophages, it was seen that the PD-L1 expression in tumor cells underwent a marked reduction, with a augmentation in T cells' anti-tumor activity^77^. Additionally, tumors deficient in LDHA displayed heightened susceptibility to anti-PD-therapy, with flow analysis revealing a significant reduction in the infiltration of Treg cells, while concurrently experiencing an increase in the number of both CD8 cytotoxic T cells and NK cells^78^. In patients with CRC who have mismatch repair gene abnormalities, blocking LDHA has been demonstrated to increase the effectiveness of anti-PD-1 therapy by downregulating MSH2 and Oct4^79^. Cyclin G2 inhibits the Warburg effect by blocking LDHA Y10 phosphorylation, thereby reversing the immunosuppressive microenvironment and halting tumor progression with a stronger anti-tumor effect when combined with anti-PD-1^77^. The potential of LDHA as a tumor therapeutic target is strongly supported by these findings. Given that tumor growth results in tissue destruction, serum LDH levels are also critical for cancer diagnosis. There has been a negative association reported in clinical trials for melanoma, urothelial carcinoma, and renal cell carcinoma between serum LDH levels and the efficacy of anti-PD-1 treatment, indicating that LDH may be used as a predictive marker for the efficacy of clinical anti-PD-1 therapy^80^,^81^. In addition, some multivariate prognostic indicators were included in serum LDH for analysis, such as the ratio of serum lactate dehydrogenase to albumin^69^. Although LDHA inhibitors are not currently the first choice for in vivo therapy due to a number of reasons, such as low success rate of selective inhibition, the rapid progress of combined immunotherapy targeting cell metabolism may lead to the emergence of better drug targeting strategies.

Single-cell sequencing of tumor infiltrates immune cells in PDAC patients showed that neutrophil-like differentiated HL-60 cells(dHL-60) overexpressed the glycolytic enzyme LDHA, along with an upregulation of the glycolysis signature. In vitro, dHL-60 cells with high LDHA directly promotes the proliferation of pancreatic cancer cells, concurrently reducing TNF-α and IFN-γ expressed by CD8+T cells, thereby amplifying the immunosuppressive and tumor-promoting effects of TANs^82^. However, it should be noted that due to the large metabolic discrepancy between tumor cells and TANs, it is not entirely possible to rely on the acute promyelocytic leukemia model HL-60 to assess the effect of TANs on immune response. He *et al.* identified serum amyloid A (SAA) as a key inflammatory mediator secreted by STAT3 pathway-activated hepatocytes, leading to resistance to PD-1 blockade therapy in HCC patients. It was found that SAA promotes the activation of the LDHA/STAT3 pathway through glycolysis, resulting in PD-L1 expression in neutrophils and the release of oncostatin M, which subsequently reduces the cytotoxic function of CD8+T cells^83^. The study proposes SAA as a promising serum biomarker for dynamically predicting the outcomes of immune therapy in clinical practice. Aldehyde dehydrogenase 1A1 (ALDH1A1), serving as a marker for cancer stem cells, is implicated in tumor growth and metastasis, contributing to drug resistance and reduced antitumor immunity. Lately, Wang et.al found that ALDH1A1 enhances the transcriptional regulation of LDHA by ZBTB7B, leading to increased lactate production and reduced anti-tumor immune cell infiltration^84^. Further, the combination of ALDH1A1 inhibitor CM2 and PD-1 mAb represents a promising therapeutic strategy to mitigate tumor immune escape. Patients with higher levels of LDH typically experience reduced benefits from ICIs. In a clinical trial on advanced metastatic melanoma, pre-treatment with BRAF and MEK inhibitors was used to decrease glycolytic activity in melanomas with BRAF mutations, leading to normalization of LDH levels in 74% of patients over eight weeks^85^. This normalization significantly improved the efficacy of subsequent ICIs therapy.

### MCT and PD-1/PD-L1 axis

Monocarboxylate transporters(MCT) are frequently overexpressed in various tumor types for lactate transport, with MCT1 and MCT4 subtypes being the most extensively studied^86^. These transporters play crucial roles in regulating intracellular and intercellular pH balance and maintaining the aggressive characteristics of the TME. Multiple small molecule inhibitors targeting MCT1 and MCT4, both selective and non-selective, have been found to activate tumor immune responses by promoting the formation of memory T cells^87^. Diclofenac effectively inhibits MCT1 and MCT4 independent of cyclooxygenase, leading to reduced lactic acid secretion by tumor cells, enhanced anti-PD-1-induced T cell cytotoxicity, sustained T cell activation, as well as delayed tumor growth and proliferation^88^. Furthermore, VB124, a highly potent inhibitor specifically targeting MCT4 suppresses tumor immune activity by promoting CD8+T cell infiltration and augmenting cytotoxicity, thus improving the efficacy of anti-PD-1 therapy in drug-resistant cancer xenograft models^89^. Consistent with previous results, Nathalie Babl demonstrated that selective inhibition of MCT4, without the necessity for concurrent blockade of MCT1, effectively counteracts lactate-induced immunosuppression and thereby augments ICB response both in vitro and in vivo within CRC models^90^. The study demonstrated that MCT4 positively regulates PD-L1 expression in TNBC by releasing lactate, which stabilizes PD-L1 through glycosylation via the WNT signaling pathway^91^. As MCT emerges as a promising molecular target, it is progressively evolving into a novel approach and strategy for immunotherapy in cancer treatment. Epigenetically, N6-methylation of adenosine (m6A) demethylases ALKBH5 adversely impacts the response to anti-PD-1 therapy in cancer, primarily through its regulation of m6A levels and gene splicing^92^. When ALKBH5 activity is inhibited through CRISPR or small molecule inhibitors, there is an increase in m6A modifications on the mRNA of MCT4. This leads to reduced MCT4 expression, lower lactate concentration, and reverses the aggregation of suppressive immune cells (Tregs) caused by lactate in the TME.

### PD-1/PD-L1 axis and glycolysis

The process of immune escape also contributes to alterations in cellular metabolism within the TME. PD-1/PD-L1 axis can directly affect glucose metabolism in pathological tissues by participating in the regulation of glycolysis-related enzymes. In vitro studies have shown that PD-1 activation on CD4+ T cells alters fatty acid metabolism, suppresses glycolysis and amino acid metabolism, and causes T cells to undergo metabolic reprogramming^93^. Their findings also revealed a notable increase in the glycolytic activity of T cells when the PD-L1 receptor was absent in mice. Blockade of checkpoint molecules PD-1, PD-L1 and CTLA4 with antibodies recovered glucose levels and improved T cell function in the TME by promoting IFN-γ production and glycolysis^94^. PD-1 imposes constraints on glycolysis, chromatin remodeling, acetyl-CoA generation and to hinder T-cell malignancy^95^. Immune checkpoint receptor PD-1, which is typically deactivated in T cell non-Hodgkin lymphoma, functions as a critical tumor suppressor in T cells^96^. Upon loss of PD-1 tumor suppressive functionality, oncogenic T cells exacerbate the activity of glycolytic remodeling and AP-1, enhancing aggressiveness and drug resistance^95^. The binding of PD-L1 to integrinβ4/integrinβ6 triggers the AKT/GSK3β and STAT3 pathway respectively, resulting in the promotion of glycolysis in cervical and bladder cancer cells^97^,^98^. Additionally, Bioluminescent imaging reveals a significant enhancement in glucose uptake and lymph node metastasis attributed to the overexpression of PD-L1^97^. A in vitro experiment has substantiated that the mediation of IFN-γ-induced augmentation of glycolytic metabolism in clear cell renal cell carcinoma necessitates PD-L1 expression^99^. In a mouse sarcoma model, PD-L1 deletion inhibits the expression of glycolytic enzymes and suppresses the mTOR signaling pathway, which lowers the rate of extracellular acidification^9^. Cui *et al.* conducted a study on the influence of PD-L1 on glucose metabolism in lung adenocarcinoma cells revealing that high PD-L1 raises the expression levels of enzymes/proteins involved in glycolysis, glucose uptake, and lactic acid generation through the PI3K/Akt pathway^114^. In addition to solid tumors, acute myeloid leukemia with elevated expression of PD-L1 promotes glycolysis through the Akt/mTOR/HIF-1α axis rendering rapid cell proliferation^100^. M Qorraj *et al.* also found that blocking PD-1 can increase the amount of glycolytic activity in monocytes, as it inhibits the main glycolytic enzymes HK2 and PKM2 in chronic lymphocytic leukemia^101^. The role of PD-1/PD-L1 improves leukemia cells' ability to metabolize glucose and fatty acid β oxidation, which helps to maintain their proliferation and survival^102^. The PD-1/PD-L1 pathway is also implicated in mesenchymal stem cell-mediated hindrance of T cell glycolysis, achieved by negatively modulating HK2 activity at the protein level, while mRNA levels are unaffected^103^. Thus, it appears that the PD-1/PD-L1 axis increases the glycolysis in tumor cells to support their proliferation, drug resistance, and invasion while suppressing the immune cells' ability to metabolize glucose, thereby weakening their anti-tumor effectiveness.

## Lactate regulates PD-1/PD-L1 axis Within TME

**Figure 4:**
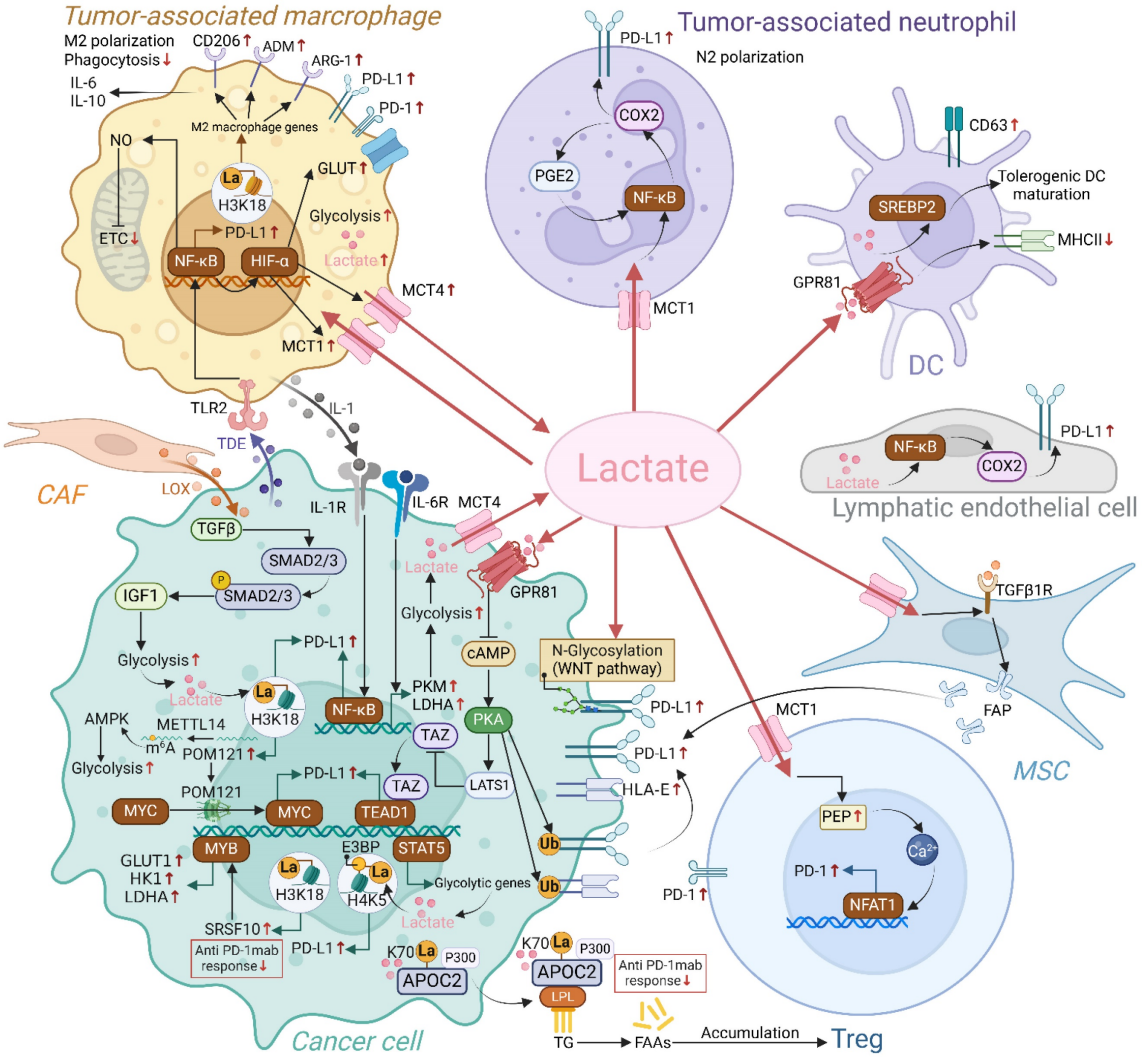
Regulation of PD-1/PD-L1 axis by endogenous and exogenous lactate in TME. Endogenous and exogenous lactate enhances PD-L1 expression in tumor cells through mechanisms including lactylation, modulation of ubiquitination, and transcriptional regulation. Additionally, lactate can upregulate PD-L1 expression in tumor cells by influencing other cells in the TME, such as mesenchymal stem cells (MSCs) and macrophages. Lactate also increases the expression of PD-L1 on neutrophils and PD-1 on Tregs, respectively. Collectively, lactate accumulation fosters a series of positive feedback loops within the immunosuppressive TME, ultimately dampening the immune response against tumor cells. (Created with Biorender.com).

Interactions between lactate and the PD-1/PD-L1 axis have been extensively studied in various cells of the TME, including TAMs, TANs, Tregs, and tumor cells (Figure 4). Epigenetic modifications and intercellular interactions involving the above regulations have participated the formation of the immunosuppressive milieu.

### Lactate and tumor-associated macrophages

TAMs constitute 15%-20% of the total mass of tumor cells in the TME and can be polarized into diverse functional phenotypes, such as M1-type and M2-type macrophages, under various stimuli^104^. M1-type macrophages can enhance inflammation and eliminate tumor cells, while M2 macrophages fuel tumor progression in primary and metastatic sites by breaching the basement membrane, fostering angiogenesis, suppressing inflammation, and bolstering immunosuppression^105^. Maintaining a high lactate level of utmost importance in sustaining the tumor-promoting activities of TAMs^106^. Activating the ERK-STAT3 signaling pathway, MCT-HIF1α signal transduction, and forming a positive feedback loop with lactate-G protein-coupled receptor 132 (GPR132) are three ways that lactate induces TAM polarization towards M2-type behaviors^107^-^109^. The latest in vitro and in vivo research has demonstrated that the overexpression of methyltransferase METTL14 in cervical cancer tissues enhances glycolytic levels via the AMPK pathway, resulting in lactate accumulation, which subsequently promotes PD-1 expression on M2 macrophage surfaces and impairs their phagocytic function^110^. Redistributed M2-type macrophages induced by lactate enhance tumor immune evasion by increasing the proportion of PD-L1+TAMs through HIF-1α-mediated signaling^111^. Tumor cells and mononuclear macrophages interact with each other through the secretion of lactic acid and IL-1β, thereby increased the PD-L1 expression on tumor cells with anti-tumor immunity inhibition in TME and mononuclear macrophage infiltration in further up-regulating the malignant loop of IL-1β synthesis and secretion^112^. Similarly, one feedforward cycle is for bladder cancer cells to reculture M2-TAM through lactic acid and promote TGF-β, which, in turn, promotes PD-L1mRNAm6A methylation via the Smad2/3 signaling pathway^113^. Apart from directly releasing lactate, tumor cells also domesticate macrophages in premetastatic lesions through the paracrine of exosomes, promoting metabolic reprogramming of macrophages, and upregulating their own PD-L1 expression via the NF-κB pathway, creating conditions for the metastasis and colonization of tumor^114^. The above studies indicate that lactic acid directly affects the polarization, phagocytosis and redistribution of TAM in TME and metastases, thus propelling the balance of tumor immunity toward the end favoring tumor progression.

### Lactate and tumor-associated neutrophils

High levels of inflammatory chemokines in TME recruit neutrophils to the tumor and domesticate them into TANs^115^. Neutrophils exhibit differentiation phenotypes resembling those of macrophages M1 or M2, called N1 and N2 subtypes respectively, with the N2 subtype playing a tumor-promoting role. Antibodies that block PD-L1 may effectively reverse the inhibitory impact of TANs on T cells' anti-tumor immunological response, indicating that TANs may have immunosuppressive effects via the PD-L1 signaling axis^116^. Mechanistically speaking, high concentrations of lactate originating from tumors may activate the MCT1/NF-KB/COX-2 pathway, which in turn can cause PD-L1 expression at high levels in TANs. Simultaneously, lactate-production increases in H^+^ concentration in TME lengthen the lifespan of PD-L1+TANs and the duration of T cell function inhibition^117^.

### Lactate and regulatory T cells

Tregs, a subset of immunoregulatory T cells, impede effector T cells' cytotoxic action against tumor cells, which facilitates the growth of tumors. The survival of Treg cells in challenging environments hinges on their flexible metabolic strategy, enabling them to utilize lactic acid in the TME as an alternative energy source to sustain self-proliferation and immunosuppressive function^38^. Treg cells ingest lactate through MCT1 to facilitate the nuclear translocation of Nuclear Factor of Activated T-cells 1(NFAT1), thereby raising their own PD-1 expression^17^. This mutually beneficial relationship between tumor-derived lactic acid, which serves as a critical energy source for Treg cells, may explain why anti-PD-1 antibody-based blocking therapies can lead to PD-1+Treg activation, resulting in immunotherapy failure^118^. High expression of MCT1/2 in Tregs and the subsequent lactate signals contribute to anti-PD-1 resistance to HCC, while MCT inhibition conformably improve the therapeutic response to PD-1 inhibitor^119^. Furthermore, lactic acid can bolster the stability and functionality of Treg cells through lactylation and promote the differentiation of CD4+T cells towards Treg^120^,^121^. Targeting MCT1 directly or inhibiting tumor acidity may disrupt this metabolic symbiosis, thereby weakening Treg cells' barrier to tumor immunity.

### Lactate and tumor cells

Lactate is able to regulate the expression of PD-L1 and the epigenetic changes made to the tumor^122^. As a receptor of lactic acid, GPR81 transduces extracellular lactate signal through G protein, thereby affecting the growth, immune regulation and drug resistance of tumor cells^123^. Further studies have found that lactic acid also can decrease the intracellular CAMP level and suppress the PKA activity by activating GPR81, thereby increasing the activity of downstream transcriptional activators, stimulating the PD-L1 promoter and prompting its expression^124^. In addition to exerting an autocrine influence on PD-L1 levels in tumor cells, lactate/GPR81 also has a paracrine impact on DC antigen presentation, which impacts tumor immune evasion^125^. It is well-established that lactic acid promotes the polarization of M2 macrophages, which are known to secrete IL-6 and IL-10. Gong *et al.* demonstrated that the IL-6/IL-6R axis enhances the expression of key glycolytic enzymes, particularly PKM and LDHA, resulting in increased lactate production. The secreted lactate stabilizes the proteins PD-L1 and HLA-E through the GPR81-cAMP-PKA signaling pathway, thereby enhancing the resistance of uveal melanoma cells to cytotoxic T cell and NK cell-mediated killing^126^. A preclinical study suggests that 3-OBA, a GPR81 antagonist, amplifies metformin's anti-tumor efficacy. The combination effectively reduces glycolysis and serum lactate levels, while the addition of PD-1 blockade further enhances anti-tumor immunity^127^. It demonstrates that lactate, through a concentration- and time-dependent manner, enhances the expression of fibroblast activation protein (FAP) in MSCs via the MCT1 and TGF-β1 signaling pathways. This activation of MSCs leads to increased migration and proliferation of gastric cancer cells, primarily mediated by the upregulation of PD-L1^128^.

Lately, Huang *et al.* have found that histone lactylation and enhanced nuclear translocation of E3BP are fostered by the accumulation of lactic acid, which is driven by STAT5 and eventually results in PD-L1 transcription in AML cell^129^. It identifies that cancer-associated fibroblasts (CAF) secrete lysyl oxidase (LOX), which activates the TGFβ signaling pathway and increases insulin-like growth factor 1 (IGF1) levels in gastric cancer cells^130^. This upregulation of IGF1 facilitates cell migration, epithelial-mesenchymal transition, and glycolysis. The accumulation of lactate leads to histone lactylation at lysine 18 on histone H3, enhancing PD-L1 transcription. Zhang *et al.* further unveiled that H3K18 lactylation directly enhances POM121 gene transcription, which is required for the nuclear localization of MYC, ultimately upregulating PD-L1 expression^131^. Impressively, blocking glycolysis with oxamate significantly strengthen the efficacy of anti-PD-1 therapy in NSCLC xenograft models. Additionally, non-histone lysine lactylation has been increasingly recognized for its role in promoting tumor progression in the context of resistance to immunotherapy. Lactate can induce lactyl-APOC2, triggering extracellular lipolysis to generate free fatty acids, which provide energy for Treg cells and support their accumulation^132^. APOC2 lactyl-K70 antibody or inhibition of lactate by FX11 enhances the sensitivity of anti-PD-1 mAb in vivo. Research identifies SRSF10 as a key gene involved in glycolysis, also correlated with resistance to anti-PD-1. In detail, SRSF10 stabilizes MYB mRNA by interacting with its 3′-untranslated region, leading to increased expression of glycolytic enzymes like GLUT1, HK1, and LDHA^133^. Lactate enters macrophages, inducing histone lactylation and activating pro-tumor transcriptional programs, further enhancing M2 macrophage function and suppressing CD8+ T cell infiltration. Targeting SRSF10 with the inhibitor 1C8 may enhance anti-PD-1 therapy effectiveness, offering a potential strategy to combat immune resistance in HCC.

## PD-L1 expression prediction: 18F-FDG PET/CT takes center stage

Patients possessing positive PD-L1 are more possibly to respond objectively to immunotherapy, and their overall and progression-free survival are increased as compared to negative patients^134^. Before starting first-line treatment, the majority of experts recommend using the immunohistochemistry (IHC) approach to track PD-L1 expression. Although IHC detection is considered the gold standard, its inability to dynamically and real-time reflect PD-L1 expression due to the requirements for sufficient biopsy tissue quality and quantity, necessitates the development of a novel non-invasive and precise imaging omics approach to assess PD-L1 expression for predicting and screening beneficial immune populations^135^. ^18^F-FDG, a radionuclide labeled compound with similar structure to glucose, is currently the most widely used PET tumor metabolism imaging agent. The Warburg effect serves as a crucial theoretical foundation for ^18^F-FDG PET/CT tumor imaging, characterized by an aberrant elevation in glucose metabolism manifested by high ^18^F-FDG uptake. In clinical practice, the utilization of metabolic standard imaging with "digital biopsy" 18F-FDG PET/CT, has become gradually widespread and holds significant value for diagnosing, staging, and monitoring the effectiveness of malignant tumors^136^,^137^.

Recently, a plethora of studies has demonstrated the utility of ^18^F-FDG PET/CT imaging in forecasting the expression of PD-L1 across a diverse spectrum of tumors, encompassing cervical cancer, ovarian cancer, gastric cancer, bladder cancer, PDAC, CRC, NSCLC, HCC, and with non-invasiveness, continuity, and reproducibility^138^. Multiple studies have found that maximum standard uptake value of lesions (SUVmax) from ^18^F-FDG PET/CT is a robust predictor of PD-L1 expression, and there is a noticeable discrepancy between individuals with high versus low PD-L1 expression^139^-^141^. However, Jreige *et al.*'s analysis of 49 NSCLC patients revealed no meaningful association between the PD-L1 expression of tumor and metabolic parameters such as SUVmax, total lesion glycolysis(TLG), metabolic tumor volume (MTV), etc^142^. Perhaps this is due to heterogeneity in the distribution of PD-L1 expression among populations and tumor tissues. Furthermore, Jreige *et al.* found a inverse relationship between the ratio of metabolic to morphological lesion volumes (MMVR) and PD-L1 expression in NSCLC patients. The relationship between ^18^F-FDG PET/CT SUV_max_, TLG, MTV, and IHC results in TME remains controversial. It is confirmed that a certain correlation between PD-L1 expression in tumor cells and metabolic uptake parameters from ^18^F-FDG PET/CT, but further investigation is warranted to figure out the details of this relationship. Interestingly, Wen *et al.* discovered that after ^18^F-FDG induction, PD-L1 was increased in a time- and dose-dependent manner^143^. Mechanistically, PD-L1 expression after DNA damage and repair is mostly up-regulated by activating of the STAT1/3 IRF1 and NF-κB/IRF3 pathway. Improved αPD-L1 mAb usage and a notable tumor growth retardation were seen when treated with a personalized combination of ^18^F-FDG stimulation and ICB. ^18^F-FDG induction will allow cold tumors with low immune activity to become hot tumors through reprogramming, making them more sensitive to ICB therapy^143^.

## Metabolism-rewriting drugs for reinvigorating antitumor immunity

Actually, natural remedies constitute a critical source of antineoplastic agents, embodying the potential for multitargeting, heightened efficacy, and minimal adverse effects. Notably, active components in natural products can directly and simultaneously target glycolysis and PD-L1 or activate anti-tumor immunity via the glycolytic pathway. For instance, Silibinin, a polyphenolic compound from milk thistle, impedes glycolytic metabolism by inhibiting HIF-1α/LDH-A, reducing PD-L1 expression on cancer cells, and enhancing immune response via the glycolytic pathway. Similarly, Ginsenoside Rh4, derived from ginseng, modulates the AKT/mTOR pathway to suppress glycolysis and reduce PD-L1 expression, thereby boosting antitumor immunity^144^,^145^. Certain bioactive components inherent in traditional Chinese medicine, such as quinones, phenolics, flavonoids, and terpenoids, display an impressive capacity to impede tumor cell proliferation, metastasis, and invasion by interfering with the glycolytic process^146^. Salvianolic acid B, for instance, inhibits glycolysis by targeting GJB2 and induces apoptosis in HCC, while simultaneously improving the efficacy of anti-PD-1 therapy by reducing T cell exhaustion^25^. Oridonin, a diterpenoid from Rabdosia rubescens, inhibits HK1, disrupts glycolysis, and reduces lactate production, which not only suppresses bladder cancer progression but also enhances CD8+ T cell cytotoxicity through PD-L1 downregulation^147^. Resveratrol, a natural polyphenol found in grapes and berries, has been shown to alter theTME in ovarian cancer by inhibiting glycolysis, thereby sensitizing tumors to anti-PD-1 immunotherapy^148^. Cannabidiol, a cannabinoid from Cannabis sativa, shifts macrophage metabolism from oxidative phosphorylation to glycolysis, which potentiates the effects of anti-PD-1 treatment in xenograft models^149^. Moreover, leveraging Chinese herbal extracts can dampen the PD-1/PD-L1 axis, rendering tumor sensitization^150^. Nevertheless, there is a paucity of research on natural small molecule drugs that concurrently target glycolysis and immune evasion. Accordingly, we posit that the combination of natural compounds with different route mechanisms offer an intriguing avenue for exploration in the realm of tumor metabolism-immunotherapy. Regarding targeted glycolysis metabolism, we mainly approach it from two aspects: one is to enhance the efficacy of anti-PD-L1/PD-1 by altering the glucose metabolism of non-tumor cells such as immune cells and tumor vascular endothelial cells; the other is to directly impact tumor cell glycolysis to augment anti-PD-L1/PD-1 efficacy, with more research currently focused on the latter^151^,^152^.

## Prospects for the future and key considerations

Metabolic plasticity of tumors moulds the tumor's biological conduct and the immune regulation in TME. Existing immunotherapies primarily enhance the destructive activity of effector T cells by blocking the interaction between PD-1 and PD-L1 on cell surfaces. As research deepens, it has been discovered that glycolytic enzymes and metabolite, lactic acid, can decrease immune cell effector function, modulate innate immune signaling, particularly in regulating the expression of PD-1/PD-L1 and monitoring the efficacy of immunotherapy. In recent years, researchers have discovered that lactic acid, regarded as an “energy currency”, serves not only as an energy source in mitochondrial oxidation but also plays a significant role in post-translational cellular modifications. It has been observed that exogenous lactic acid, acting as a driving force for histone lysine lactylation, governs downstream gene expression and facilitates the M1/M2 polarization in macrophages. This striking revelation intertwines cellular metabolism with gene regulation, illuminating the indispensable role of lactylation in the intricate tapestry of pathological and disease processes. However, the involvement of tumor-derived or other stromal cell-generated lactate in regulating or modifying pertinent genes in anti-tumor immunity remains enigmatic. There is a likelihood that lactylation contributes to regulating crucial proteins, and interrupting lactylation suppression could present a novel strategy to fortify the effectiveness of immunotherapy. In clinical practice, there is compelling rationale for utilizing ^18^F-FDG PET/CT as a means to expediently forecast PD-L1 levels and identify potential beneficiary groups. Metabolic rewriting drugs to activate tumor immunity is an ever-expanding realm with ongoing advancements, and the application of combined multiple natural drugs necessitates further basic studies and clinical trials to validate their efficacy. Targeting glycolysis in combination with anti-PD-1/PD-L1 have shown alluring prospect, and we have compiled a comprehensive collection of preclinical and clinical trials for further in-depth investigation (Table 1).

In general, the overarching effects of the tumor immune response typically arises from the dynamic interplay between tumor cells and neighboring cells, involving metabolic reprogramming and immune checkpoint signaling pathways. A thorough understanding and appropriate use manipulation of the crosstalk between these elements in the tumor immune microenvironment have the potential to boost the efficacy of tumor immunotherapy and address the low response rate of immunotherapy. It should be noted that complete suppression of glycolysis seemingly not be feasible. The fact that cancer cells and immune cells share many similar metabolic needs may render them equally susceptible to metabolic regulation. This implies that inhibition of glycolysis may potentially impair T cells and DCs function, leading to the loss of their glycolytic-dependent functionalities^153^. An innovative study by Sahil Inamdar and collaborators demonstrates how systemic glycolysis inhibitors can block the glycolytic process in cancer cells, while concurrently supplying antigen-presenting cells with phagocytic particles derived from metabolites. This strategy allows for the development of DC-based immunotherapies, even when glycolysis inhibitors are used^154^. Identifying specific targeted therapeutics at optimal dosages to target tumor cells without compromising immune-mediated killing effect is paramount. While metabolic drug combination immunotherapy has been utilized in clinical trials, a deeper comprehension of the link between tumor immune escape mechanism and cell metabolism is required to fully unlock the potential of combination therapy and boost the anti-tumor effectiveness.

## Figures and Tables

**Figure 1:**
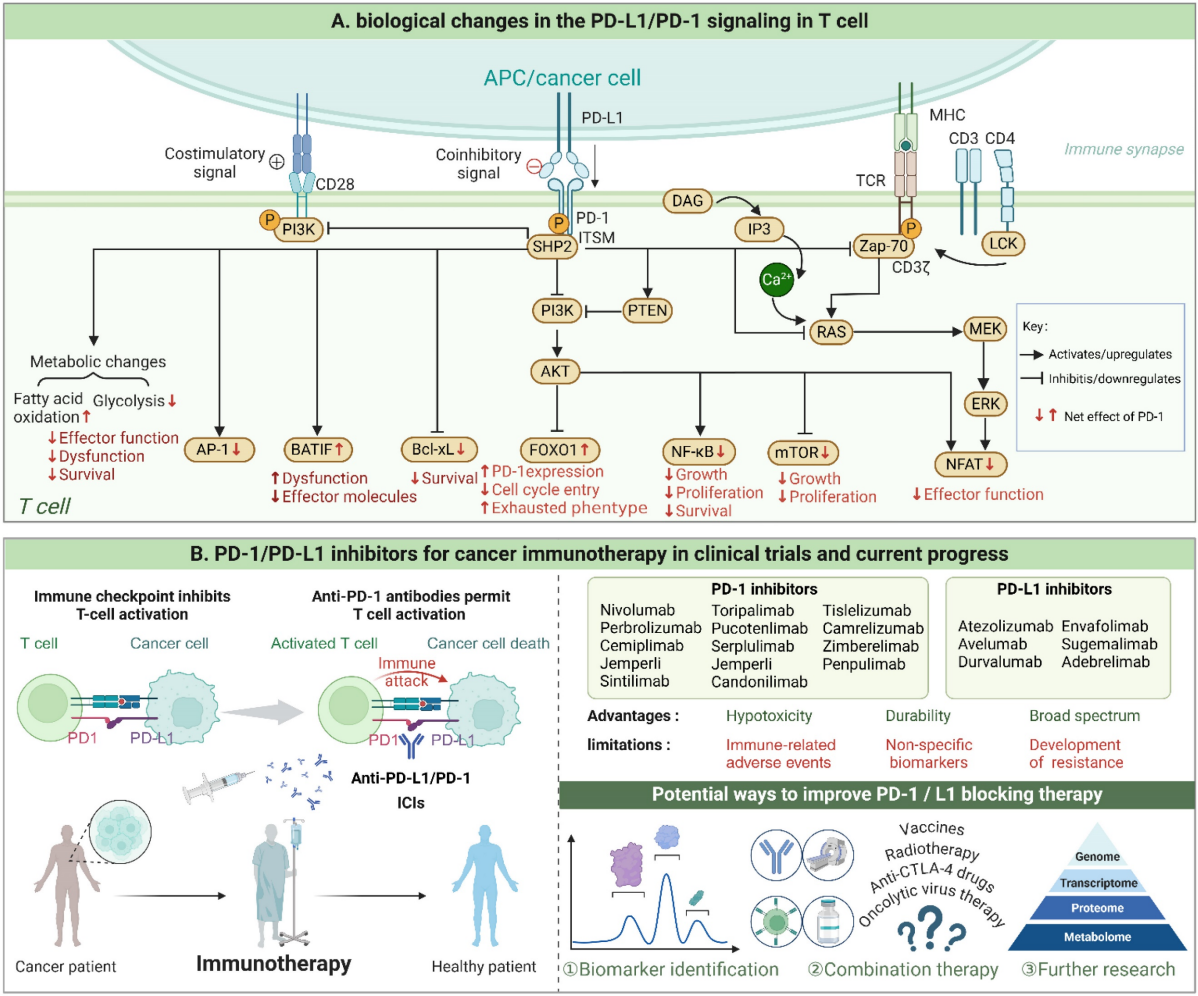
PD-1/PD-L1 signaling in T cell and progress of PD-1/PD-L1 inhibitors. When PD-1 on T cells binds to PD-L1 on tumor cells, a cascade of intracellular events is initiated, culminating in the suppression of immune responses. This binding triggers the phosphorylation of the ITSM motif, followed by the recruitment of protein tyrosine phosphatases such as SHP-2. These enzymes then dephosphorylate critical signaling proteins necessary for T cell activation. As a result, signaling pathways crucial for various cellular processes—specifically, RAS/MAPK/ERK and PI3K/PTEN/AKT/mTOR—are inhibited. This inhibition also disrupts TCR activation and alters metabolic pathways, favoring fatty acid oxidation over glycolysis, which further suppresses T cell proliferation and survival. Consequently, cytokine production is reduced, T cell effector functions are diminished, and a transcriptional program that leads to T cell exhaustion is promoted. (Created with Biorender.com).

**Table 1:** Preclinical and clinical trials of drugs targeting glycolysis with PD-1/PD-L1 inhibitors in anti-tumor therapy.

Preclinical/Clinical trials	Combined treatment (Glycolytic metabolism targeting+PD-1/PD-L1 inhibitors)	Mechanism of action on glycolysis/lactate	Targeting models/patients	Outcome from trial	Refs.
Preclinical trials	BAY-876(GLUT1 inhibitor)+anti-PD-1	GLUT1	Tumor-bearing murine models (GBM)	The use of BAY-876 sensitizes tumors to immunotherapy and works synergistically with anti-PD-1 therapy.	(22)
Preclinical trials	Lonidamine(HK inhibitor)+anti-PD-1	HK2	Tumor-bearing murine models (GBM)	Combined treatment with an HK inhibitor and an anti-PD-1 remarkably augments the anti-tumor efficacy of ICB in glioblastoma.	(32)
Preclinical trials	PFK-015+anti-PD-1	PFK	Tumor-bearing murine models (ESCC)	The combination of PFK015 and anti-PD-1 exhibits stronger tumor inhibition function.	(40)
Preclinical trials	PKM2 knockdown +anti-PD-1	PKM2	Tumor-bearing murine models (PDAC)	Knockout of PKM2 gene enhances the antitumor effect of anti-PD-1 blocking in PDAC.	(58)
Preclinical trials	DY131(selective ESRRG agonist)+anti-PD-1	PKM2	Tumor-bearing murine models (ESCC)	The administration of DY131 reduced lactic acid production in tumor cells and significantly potentiated the anti-tumor effect of anti-PD1.	(60)
Preclinical trials	Dichloroacetate(PDK1 inhibitor)+anti-PD-L1	PDK1	Tumor-bearing murine models (ovarian cancer)	The co-treatment of dichloroacetate and anti-PD-L1 synergistically improves overall survival rate in ovarian cancer mice.	(67)
Preclinical trials	PDK3-knockdown+antiPD-1	PDK3	Tumor-bearing murine models (CRC)	The combination of PDK3 inhibition with PD-1 blockade significantly enhances the survival rate in mouse tumor models.	(68)
Preclinical trials	JX06(PDK1 inhibitor)+anti-PD-1	PDK1	Tumor-bearing murine models (PDAC)	The co-treatment of JX06 and anti-PD-1 exhibited a pronounced tumor-suppressive effect in PDAC.	(70)
Preclinical trials	LDHA knockdown +anti-PD-1	LDHA	Tumor-bearing murine models (CRC)	Knockout of the LDHA gene enhances the efficacy of PD-1 blockade in a pMMR CRC transplantation model by downregulating MSH2 and Oct4.	(79)
Preclinical trials	Cyclin G2+anti-PD-1	LDHA	Tumor-bearing murine models (Gliomas)	Cell cycle protein G2 can enhance the blocking effect of PD-1 and exert a strong anti-tumor immune response.	(77)
Preclinical trials	VB124+anti-PD-1	MCT4	Tumor-bearing murine models (HCC)	Combining VB124 enhances the therapeutic efficacy of anti-PD-1 immunotherapy in hepatocellular carcinoma and prolongs the survival time of mice.	(89)
Preclinical trials	MCT4 inhibitor+anti-PD-L1	MCT4	Tumor-bearing murine models (CRC)	Combined application of single MCT4 inhibitor and ICB can potentiate inhibition of tumor growth and prolong survival.	(90)
Preclinical trials	Resveratrol+antiPD-1	PKM2 and GLUT1	Tumor-bearing murine models (ovarian cancer)	Enhanced anti-tumor effectiveness observed with the combination of resveratrol and antiPD-1.	(148)
Preclinical trials	Cannabidiol+anti-PD-1	Shift metabolic process into glycolysis in macrophages	Tumor-bearing murine models (CRC)	Cannabidiol mediated macrophage metabolic plasticity enhances the response of xenografted mice to anti-PD1 immunotherapy.	(149)
Preclinical and clinical trials	Osimertinib+anti-PD-1	GAPDH	CRC patient (Stage IIIB colon cancer with lung metastasis) and mouse model	Low-dose osimertinib can enhance the effectiveness of anti-PD-1 in immunotherapy for colorectal cancer by reducing endothelial lactate secretion.	(151)
Preclinical trials	FX-11+anti-PD-1	LDH	Tumor-bearing murine models (TNBC)	The suppression of LDH augment the efficacy of anti-PD-1 immunotherapy.	(91)
Preclinical trials	Bicarbonate+anti-CTLA-4 or anti-PD-1	Lactate	Tumor-bearing murine models (B16 melanoma)	The coadministration of bicarbonate therapy to counteract tumor acidity alongside anti-CTLA4 or anti-PD1 agents exhibited enhanced antitumor responses.	(16)
Preclinical trials	3-OBA+Metformin+anti-PD-1	Lactate/GPR81	Tumor-bearing murine models (CRC)	The combination inhibition of lactate/GPR81 and the PD-1/PD-L1 pathway amplifies the activity of CD8+T cells and significantly boosts the effectiveness of metformin in combating tumors.	(127)
Preclinical trials	NAT10 knockdown+anti-PD-L1	Nat10/ac4C/Foxp1/GLUT4 and KHK	Tumor-bearing murine models (cervical cancer)	The deficiency of the NAT10 gene enhances PD-L1 blocking-mediated tumor regression *in vivo* cervical cancer.	(26)
Preclinical trials	GJB2 knockdown+anti-PD-1	HIF-1/GLUT1	Tumor-bearing murine models (HCC)	Inhibition of GJB2 enhances the sensitivity of anti-PD1 therapy.	(25)
Preclinical trials	2-DG+anti-PD-1	Glycolysis driven by 5-HT	Tumor-bearing murine models (NSCLC)	The glycolysis inhibitor 2-DG significantly boosts anti-tumor efficacy of anti-PD-1 therapy.	(27)
Preclinical trials	Citalopram+anti-PD-1	GLUT1	Tumor-bearing murine models (HCC)	Citalopram is effective in liver tumors with high glycolysis and potentiates anti-PD-1 treatment.	(28)
Preclinical trials	CXCR1/2-knockout+anti-PD-1	IL-8/CXCR2/HK2	Tumor-bearing murine models (gastric cancer)	CXCR1/2 depletion enhances the effect of anti-PD-1.	(36)
Preclinical trials	CircFAM13B+anti-PD-1	PKM2	Tumor-bearing murine models (bladder cancer)	The overexpression of CircFAM13B enhances the effectiveness of anti PD-1 therapy.	(61)
Preclinical trials	CircRHBDD1 inhibition+anti-PD-1	CircRHBDD1/YTHDF1/PIK3R1	Tumor-bearing murine models (HCC)	Inhibition of CircRHBDD1 and combined anti-PD-1 treatment significantly suppress tumor growth and improve overall survival rate	(62)
Preclinical trials	ALKBH5 inhibitor+GVAX+PD-1 Ab	MCT4	Tumor-bearing murine models (CRC)	ALKBH5 inhibitor enhances efficacy of the combination with GVAX and PD-1 Ab	(92)
Preclinical trials	MCT inhibitor+anti-PD-1	MCT	Tumor-bearing murine models (HCC)	MCT inhibitor promote the curative effects of anti-PD-1 therapy.	(119)
Preclinical trials	Oxamate+anti-PD-1	LDHA/Lactylation	Tumor-bearing murine models (NSCLC)	Glycolysis blockade tellingly boosts anti-PD-1 efficacy.	(131)
Preclinical trials	APOC2 lactyl-K70 antibody or FX11+anti-PD-1	LDHA/Lactate	Tumor-bearing murine models (NSCLC)	APOC2K70-lac antibodies sensitize NSCLC to anti-PD-1 therapy.	(132)
Preclinical trials	1C8(SRSF10 inhibitor)+anti-PD-1	SRSF10/MYB/glycolysis	Tumor-bearing murine models (HCC)	1C8 inhibition impeded tumor progression and enhances anti-PD-1 treatment response.	(133)
Clinical trials (NCT04328740)	TP-1454 or+ipilimumab/nivolumab	PKM2	Patients with advanced solid tumors	Phase 1, active.	(55)
Clinical trials (NCT02968303)	BRAF and MEK inhibitor+anti-PD-1	Glycolysis/LDH	Metastatic melanoma patients	Phase 2, active.	(85)

## Abbreviation

5-HT: 5-hydroxytryptamine
ALDH1A1: Aldehyde dehydrogenase 1A1
ApoA1: apolipoprotein A1
CAF: cancer-associated fibroblasts
CRC: colorectal cancer
DC: dendritic cells
ESCC: Esophageal squamous cell carcinoma
ESRRG: estrogen-related receptor gamma
FAP: fibroblast activation protein
FAP: Fibroblast activation protein
FN1: fibronectin 1
G-6-P: glucose-6-phosphate
GCMSCs: gastric cancer mesenchymal stem cells
GJB2: gap junction protein beta-2
GPR81: G protein-coupled receptor 81
GPR132: G protein-coupled receptor 132
HCC: hepatocellular carcinoma
HK: hexokinase
ICB: immune checkpoint blockade
ICIs: immune checkpoint inhibitors
IHC: immunohistochemistry
ITSM: immunoreceptor tyrosine-based switch motif
LDH: lactate dehydrogenase
LDHA: lactate dehydrogenase
LOX: lysyl oxidase
MCT: Monocarboxylate transporters
MSC: mesenchymal stem cells
NGF: Nerve growth factor
NK: natural killer cell
NFAT1: Nuclear Factor of Activated T-cells 1
PDK: Pyruvate dehydrogenase kinase
PD-1: programmed death receptor
PDAC: pancreatic ductal adenocarcinoma
PFK: Phosphofructokinase
PK: pyruvate kinase
PKM: pyruvate kinase M
SUVmax: maximum standard uptake value of lesions
SAA: serum amyloid A
TLG: total lesion glycolysis
MTV: metabolic tumor volume
MMVR: the ratio of metabolic to morphological lesion volumes
TAM: tumor-associated macrophage
TANs: tumor-associated neutrophils
TME: tumor microenvironment
TNBC: Triple-negative breast cancer
Tregs: regulatory T cells
VDAC: voltage-dependent anion channels
m6A: N6-methylation of adenosine
IGF1: insulin-like growth factor 1
NSCLC: non-small cell lung cancer
MHC: major histocompatibility complex
